# Experimental Study of Three AlSi10Mg Cellular Structures with Triply Periodic Minimal Surface (TPMS) Topology Subjected to Bending Loading and Identification of Root Aspects of Possible Premature Failure

**DOI:** 10.3390/ma19122669

**Published:** 2026-06-21

**Authors:** Katarina Monkova, Peter Pavol Monka

**Affiliations:** Faculty of Manufacturing Technologies with a Seat in Presov, Technical University of Kosice, Sturova 31, 080 01 Presov, Slovakia; peter.pavol.monka@tuke.sk

**Keywords:** cellular structures, SLM technology, bending, failure, AlSi10Mg, fracture surface

## Abstract

**Highlights:**

**What are the main findings?**

- The study combines comparative bending experiments, preliminary manufacturing optimization, and fracture surface/EDX analysis for three relatively less investigated TPMS topologies in AlSi10Mg.
- The manufacturing conditions of thin-walled TPMS cellular structures greatly influence premature failure.
- Within the investigated range of volume fractions, the load-bearing capacity, effective stiffness, and absorbed energy increased, while the Fischer–Koch S topology showed the most balanced response overall and the Schoen F-RD topology achieved the highest nominal bending stress.

**What are the implications of the main findings?**

- Inappropriately delivered laser energy during SLM production of thin-walled cellular structures caused structural heterogeneity.
- Incoherent or semi-coherent bonding of unmelted particles to the matrix reduces resistance to crack initiation and propagation.
- The present three-point bending configuration should be understood as a comparative benchmark for flexural response of sandwich-type TPMS specimens rather than a direct validation of complex industrial components subjected to combined loading.

**Abstract:**

The manuscript deals with the bending behavior of beams with relatively less investigated cellular topologies based on triply periodic minimal surfaces (TPMSs). Three types of sandwich-type specimens (namely Schoen IWP, Fischer–Koch S, and Schoen F-RD) with five different volume fractions of 10, 15, 20, 25, and 35% (±1%) made of aluminum alloy AlSi10Mg by selective laser melting (SLM) technology were investigated. Three-point bending tests were performed at room temperature on a Zwick/Roell 1456 universal testing machine. The force–deflection dependences were plotted, while in addition to nominal stresses, the effective flexural stiffness and energy absorption to failure were evaluated to compare the properties of the investigated cellular beams. In the preparatory phase, critical aspects of possible premature failure of the samples with the smallest and highest selected volume fractions were addressed, while the manufacturability and fracture surfaces of the samples were assessed in order to improve the input conditions of the setup. By comparing the results obtained in the experimental testing in the second phase, it was found that the highest nominal bending stresses were achieved by the Schoen F-RD structure (although not significantly higher than Fischer–Koch S), but in terms of stiffness and amount of absorbed energy, the Fischer–Koch S structure showed the highest values. The improvement of input parameters led to an increase in the achieved nominal bending stresses by at least 100 MPa for all types of investigated structures compared to the first phase. The combined use of preliminary SLM process optimization, bending tests, and fracture surface/EDX analysis made it possible to relate the flexural response of the investigated TPMS topologies to manufacturing-related defects and premature-failure mechanisms in thin-walled AlSi10Mg cellular structures. The presented specimen configuration is intended as a comparative experimental benchmark for flexural performance of sandwich-type TPMS beams under quasi-static loading.

## 1. Introduction

Nowadays, the fastest developing technologies can be considered additive technology, which not only provides many opportunities for chip-free production of parts from various materials but also new challenges. The possibility to produce complex-shaped parts is one of them. Complexity can be introduced not only by outside geometry but also by the inside topology of a body. The additive production approach has allowed stochastically distributed pores in the lightened bodies to be replaced by a regular cellular structure, the properties of which are far better controlled, and the behavior of the body is thus more predictable compared to components containing randomly distributed cavities in the core of the body.

Porous or cellular structures are often used as a lightweight core that fills a solid body in the form of a shell or that separates two solid outer layers to form a sandwich panel. As in the case of an I-beam, such structures may provide a higher flexural stiffness-to-weight ratio than classic solid beams with square cross-sections. Therefore, the use of cellular structures is advantageous when weight savings are important while maintaining sufficient stiffness. They can be considered for a wide range of lightweight engineering concepts, including applications in aerospace, aviation, or marine industries [1,2]. At the same time, the direct applicability of a specific cellular design always depends on the actual component geometry, boundary conditions, and loading regime; therefore, benchmark tests on simplified specimens remain an important first step before broader component-level validation.

**Figure 1:**
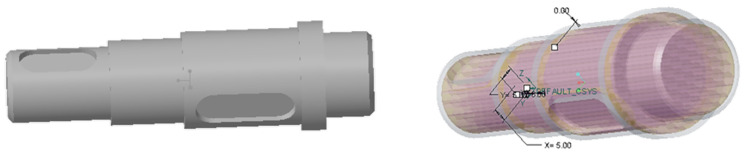
Shaft with a lightweight structure as its core.

In the automotive industry, lightweighting concepts may also be considered for components such as, e.g., gear-related parts or shafts (Figure 1).

However, a simple uniaxial tensile or compressive test is not sufficient in many cases to understand all aspects of a material’s behavior and properties. A more comprehensive approach to property evaluation is the bending test to failure, which involves combined stressing of the specimen, including tension, compression, and shear [3,4]. Therefore, bending tests are often used to assess the structural response of materials and cellular architectures under a controlled loading configuration. The results of bend testing provide useful comparative information for assessing flexural load transfer, stiffness, and failure initiation, while the relevance to a specific engineering application must still be verified under the corresponding service loading conditions [5].

This example, however, serves only as an illustration of the broader engineering motivation, as real components are mechanically more complex than the configuration investigated in the present work because, while in service, they are typically subjected to combined and dynamic loading.

## 2. State of the Art

Many scientists and specialists have already dealt with the behavior of the bending properties of beams made of available materials, as well as porous or sandwich beams, but the study of the behavior of beams with porous structures with regularly distributed cells of complex shapes under bending loading is still an unexplored area. Only several studies are accessible to evaluate the bending behavior of porous structures, but in most cases, they are made of plastic. The following studies are mentioned as examples.

Horn et al. [6] investigated prismatic Ti6Al4V bars produced via electron beam melting (EBM), incorporating rhombic dodecahedral unit cells of varying sizes and relative densities, that were subjected to four-point bending tests. Although their findings generally aligned with Gibson and Ashby’s power-scaling models, the practical application of these models as design tools is constrained by machine resolution—particularly for structures with small pore sizes necessary for bone ingrowth.

The structural durability of 3D-printed meta-sandwiches through quasi-static bending and low-speed impact tests was assessed by Sarvestani [7], employing both Finite Element simulations and experimental methods. Analytical formulas were developed to elucidate the failure mechanisms within sandwich structures. The study determined that core topology and geometrical parameters significantly influenced both the failure mechanism and energy absorption characteristics of meta-sandwich structures; notably, iso-max meta-sandwich configurations demonstrated superior quasi-static and dynamic energy absorption during impact scenarios.

The team led by Tüzemen [8] investigated the mechanical properties of functionally graded porous structures, examining three distinct unit cell types—rhombuses, cubes, and octahedroids—as well as three thicknesses and three unit sizes. The study aimed to determine the relationship between porosity and the elastic modulus of the cellular structure. Specimens were fabricated by laser melting Ti-6Al-4V powder beds and subsequently subjected to three-point bending tests following heat treatment. Experimental results demonstrated a strong correlation with theoretical calculations.

Araújo et al. [9] sought to analyze and assess the impact of core geometry on the flexural properties of three cell configurations—regular honeycombs, lotus honeycombs, and hexagonal honeycombs with plateau edges—each evaluated at four relative densities. Flexural properties were assessed through three-point bending tests using both numerical and experimental approaches, complemented by modeling for two materials—polylactic acid and pure aluminum—in all three configurations. The findings revealed significant variation attributed to geometric configuration and indicated a pronounced dependence on the relative density regarding energy absorption, stiffness, and bending strength.

Investigations of Muhammad [10] related to bendability enhancement of an age-hardenable aluminum alloy was realized using a series of wrap–bend tests, with emphasis on understanding the relationship between the microstructure, the nature of plastic deformation, and fracture behavior. The composite AA6016X alloy consisted of a central core of AA6016 sandwiched between 100 μm thick clad layers of AA8xxx series aluminum alloy, which was processed using thermomechanical roll-bonding. It was shown that the bendability of monolithic AA6016 alloy is limited due to the formation of severe surface undulations and surface cracking, which are associated with the heterogenous nature of slip that concentrates into 5–15° misoriented coarse slip bands of very high dislocation content in the order of 1014/m^2^, as well as intense shear bands originating from surface low cusps in the form of mutually orthogonal transgranular bands.

Friedman [11] studied bendability of two Al-Mg-Si heat-treatable alloys and compared this with the performance of a non-heat-treatable Al-Mg alloy. Bending failure in the alloys was based on a surface-roughening, or orange peel, process where the outer surface grains separated and produced depressions on the surface of the material. The authors stated that these depressions acted as notches that increased local stresses and eventually caused failure. The fracture was intergranular in nature, with a jagged crack progressing through the thickness of the material. Critical elements regarding natural aging, artificial aging deformation, and composition were discussed within the manuscript.

Similarly, in their research, Gullapali & Massod [12] studied 2D cellular lattice structures fabricated from ABS material by the Fused Deposition Modeling (FDM) technique, while employing a Stratasys Dimension 1200 ES machine. They investigated the beams of lattice structures from rectangular cross-sections with diverse unit cell arrangements. Experimental investigations under three-point bending were conducted to evaluate the flexural strength and Young’s modulus of cellular lattice structures. The findings demonstrated that lattice structures configured with triangular and honeycomb geometries exhibited superior maximum bending strength.

Choi et al. [13] studied flexural properties of ABS specimens with honeycomb structures made by FDM 3D printing. They found that fracture surface roughness increased with temperature from 30 to −50 °C after examining surfaces.

Chen et al. [14] analyzed porous beams in bending using Timoshenko beam theory, with graded modulus of elasticity and density across the thickness. Results showed maximum deflection rose with a higher porosity coefficient and slenderness ratio, while normal stress distribution shifted from linear to non-linear as these parameters increased.

In addition to being used as complete cellular architecture, TPMS-based geometrical concepts are increasingly recognized as useful local design features for improving load transfer, smoothing stress trajectories, and reducing critical stress concentrations in lightweight structures. Recent research on architected materials, graded lattices, and TPMS-based structural optimization suggests that such geometry-driven modifications can contribute significantly to the mechanical efficiency and damage tolerance of additively manufactured cellular systems [15,16,17]. Within this broader engineering context, the present study addresses a more specific question, namely how selected relatively less investigated TPMS topologies manufactured from AlSi10Mg by SLM behave under bending and how their response is influenced by manufacturing-related factors.

There were perhaps only a few other studies that were found regarding the testing of bending or flexural properties of regularly distributed cellular structures and their fracture surfaces, such as [18,19,20,21,22,23], but after a thorough search and based on the available studies and findings of the authors, the behavior of selected types of porous materials with a complex topology of cells made of aluminum alloy by SLM technology under bending loading has not been investigated in this way, or only to a very small extent. The present work focuses on the flexural response of sandwich-type TPMS specimens manufactured from AlSi10Mg by SLM and combines comparative bending experiments with preliminary process optimization and fracture surface/EDX analysis in order to relate structural performance to manufacturability and failure-relevant defects.

## 3. Materials and Methods

### 3.1. Samples Design

The properties of components based on cellular materials are determined by several factors, the most important of which include the basic material from which the structure is made, as well as the topology of the cell determined by size and geometry. Another crucial factor is the volume fraction of a cellular structure, which indicates the proportion of solid material within the total volume of the specimen. This ratio can be determined using Equation (1) [24,25]:

(1) $$V_{f}=\;\frac{V_{S}}{V_{T}}\;\times 100\;(\%),\;$$

where *Vs* is a volume of the solid material (mm^3^), and *V_T_* (mm^3^) is the total volume of the specimen.

**Figure 2:**
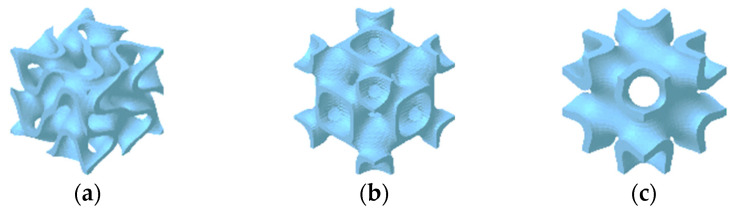
The basic cells of the investigated structures: (**a**) Fischer–Koch S; (**b**) Schoen F-RD; (**c**) Schoen IWP.

For the presented research, the sandwich-type samples with three types of structures—Fisher–Koch S, Schoen F-RD, and Schoen IWP—were chosen as interesting types of cellular materials that are rarely used but whose properties could be promising for various types of applications. These types of structures were chosen as representatives of complex structures not only because they meet the requirement of self-support, which is very important in their production if they were to be applied in the core of the component (where the removal of the supporting structures would be problematic), but also because of the fact that research on the behavior of these structures and their mechanical properties is still not sufficient. The basic cells of the structures are shown in Figure 2.

The structures are part of a group of so-called triply periodic minimal surfaces (TPMSs). TPMSs are surfaces that locally minimize the surface area of the boundary designated, so that the average curvature at each point on the surface is zero and can be further categorized into ligament/skeletal/solid networks or sheet networks. They can be accurately described by the mathematical Equations (2)–(4) [26].

Fischer–Koch S:

(2) $$f\left(x,y,z\right)=cos\left(2x\right)sin\left(y\right)cos\left(z\right)+cos\left(x\right)cos\left(2y\right)\sin\left(z\right)+\sin\left(x\right)\cos\left(y\right)\cos\left(2z\right)\;\times \;z$$

Schoen F-RD:

(3) $$f(x,y,z)\;=4\cos\left(x\right)\cos\left(y\right)\cos\left(z\right)-\left[\cos\left(2x\right)\cos\left(2y\right)+\cos\left(2y\right)\cos\left(2z\right)+\cos\left(2z\right)\cos\left(2x\right)\right]$$

Schoen IWP:

(4) $$f\left(x,y,z\right)=\left[\cos\left(2x\right)+\cos\left(2y\right)+\cos\left(2z\right)\right]-2\left[\cos\left(x\right)\cos\left(y\right)+\cos\left(y\right)\cos\left(z\right)++\cos\left(z\right)\cos\left(x\right)\right]$$

**Figure 3:**
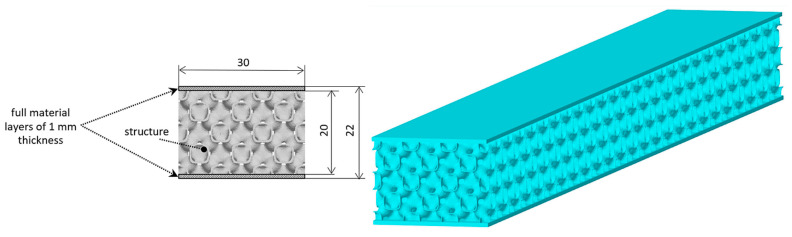
Cross-sectional view of a Shoen F-RD bending specimen with basic dimensions and a view of the entire virtual 3D model.

Within this study, 3D models of the specimens with five volume fractions *V_f_* = 10, 15, 20, 25, and 35% (±1%) were generated in Rhinoceros^®^ 8 software using Equations (2)–(4) with an additional Grasshopper tool. The specimen core was filled with a basic cell of a given topology, and the dimensions of which were 10 × 10 × 10 mm. The basic cell was evenly patterned in all three orthogonal directions of *x*, *y*, and *z*, so the number of cells in each direction was 3, 25, and 2, respectively. The total dimensions of the samples, 30 × 250 × 22 mm, were not only adapted to the possible parameters of the 3D printer and requirements of the testing equipment used but took into account the testing method as well. The different target volume fractions were obtained by controlling the wall thickness of the sheet-based TPMS geometry in the virtual model, while keeping the TPMS topology, the unit cell size, the number of repeated cells, and the external dimensions of the specimen constant. The resulting volume fraction of each specimen was then determined from the final 3D model as the ratio of solid volume to total specimen volume. With this in mind, the thin-walled structures were designed so that the beam length was dominant, and the cell wall deformation did not occur primarily due to the pressure of the testing machine thumb. To prevent damage to the thin walls of the structures due to the pressing of the push thorn of the testing machine before the sample fails under bending loads, the top and bottom of the porous structure with dimensions of 30 × 250 × 20 mm was covered with a continuous 1 mm thick skin (continuous solid layer) of the same material, as shown in Figure 3. The reason for using these skins/layers was also due to the fact that porous components usually cannot be used without a surface shell in common technical practice (mainly due to connection with other components) and therefore the lightweighting structure (or pores) provides relief only in the core of the component. In the present context, the term sandwich-type specimen denotes the tested beam-like configuration consisting of a TPMS cellular core and two continuous outer skins, while thin-walled refers to the morphology of the TPMS cell walls manufactured by SLM.

### 3.2. Material and Specimens Production

The experimental specimens were manufactured with the idea of using the advantages of porous structures and the prospective goal of implementing such lightweight components in the aviation or automotive industry; an aluminum alloy AlSi10Mg alloy (EU: 3.2381, USA: A 360; ASTM F3318-18) was proposed as a material for the production of samples and was provided in the form of powder. The alloy is apposite for an extensive range of industrial applications. It is a light alloy with a relatively low density (2.66 g/cm^3^) compared to other structural materials, which exhibits good alloying properties and thermal and electrical conductivity. The basic tensile properties given by the powder manufacturer after solid body production are defined by the material sheet [27]:

- Ultimate tensile strength Rm = 449 ± 10 MPa (vertical).
- Yield strength Rp_0.2_ = 236 ± 5 MPa.
- Young’s modulus E = 72 ± 5 GPa.
- Elongation at break (as built) 7 ± 2%.

The chemical composition of the aluminum alloy is shown in Table 1 [27].

The specimens were manufactured by SLM technology employing a 3D printer EP-M250 (E-Plus-3D; Hangzhou, Zhejiang, China) with the proprietary Eplus3D printing software for EP-250, using fiber laser power 500 W with Argon gas supply, and a flow rate of 40 m/s. The cellular structures in the samples’ cores were printed without additional support since they were self-supporting.

For a preliminary phase, six samples were produced, one per each specimen of all three types with volume factions 10 and 35%, and evaluated from the technological conditions’ settings perspective. After a preliminary parameter setting phase, in which critical points of failure of sample production were identified, samples with the same three types of structures as in the preliminary phase were produced under new input conditions, but with five volume fractions of 10, 15, 20, 25, and 35%. For reasons of repeatability, identification of outliers, and statistical evaluation of the results, three samples with the same topology (structure and volume fraction) were 3D printed, so that a total of 45 samples were subjected to the three-point bending test in the second phase.

Additional manufacturing settings will be specified in the next part of the manuscript with respect to the sample fabrication/testing phase.

### 3.3. Experimental Testing and Evaluation

**Figure 4:**
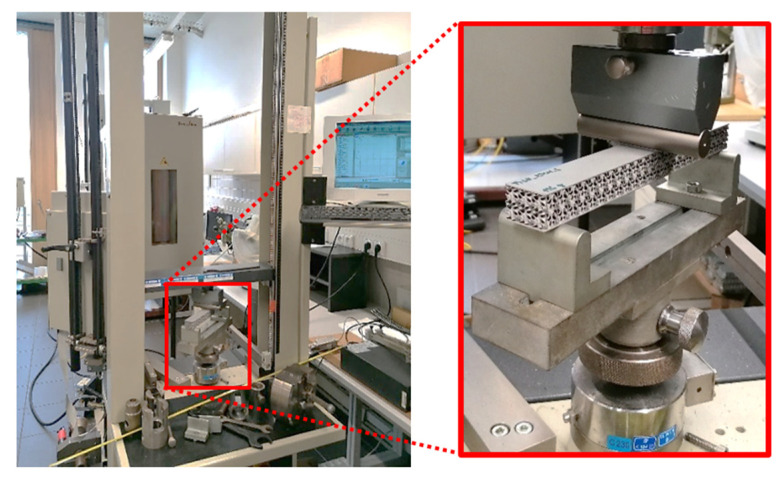
The experimental setup with a detailed view of the Fischer–Koch S specimen under loading.

A three-point bending test was employed in the investigation to study the complex cellular structures behavior until failure, as it involves multiple forms of failure compared to tensile or compression tests. The tests were performed according to ISO 7438:2020 [28] at an ambient temperature of 22 °C and a humidity of 55%, using a ZWICK 1456 testing machine (Ulm, Germany) with a load capacity of 20 kN. The samples were positioned and centered on supports with a span of 220 mm so that the push thorn acted perpendicular to the continuous layer of material covering the specimen structure (Figure 4).

At the beginning of the test run, the specimen was loaded at a rate of 1 mm/min, with the data required to calculate the bending stiffness recorded in the initial stage using testXpert III software, version 1.5. This crosshead speed was set to a deflection of 1 mm, then the speed was increased to 20 mm/min and continued until the specimen failed. Both loading rates corresponded to quasi-static testing conditions, while their difference was motivated by the different objectives of the two evaluations. The lower speed of 1 mm/min was used to obtain a more stable and accurate record of the initial load–deflection response for stiffness determination, whereas the speed of 20 mm/min was used in the main bending-to-failure tests as a practical quasi-static rate for comparative testing of all specimens.

#### 3.3.1. Fracture Surface Evaluation

**Figure 5:**
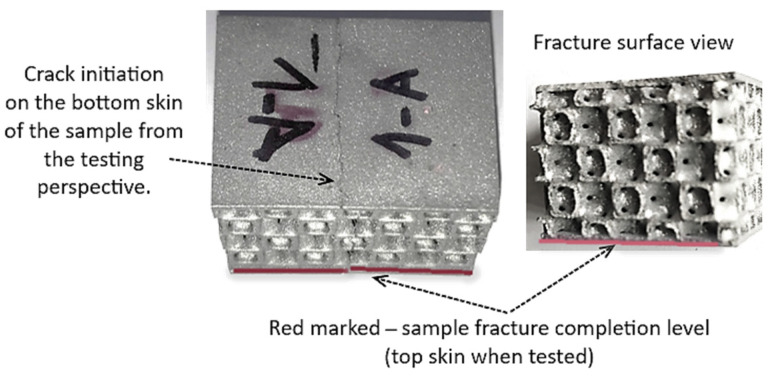
An example of analyzed sample subjected for fractographic and EDX analyses.

Damaged porous samples (six pieces, with the lowest and highest volume fraction of 10 and 35% of all three types of structure) that had been manufactured and experimentally tested within the preliminary phase of the research were subjected to fractographic and EDX analyses using a Vega 3 Tescan electron microscope (Brno, Czech Republic) and a Bruker analyzer (Bruker AXS Microanalysis GmbH Berlin, Germany) with Esprit software (version 1.9). The samples with primary initiated cracks due to bending loading on the bottom skin of the specimen (relative to the test position) were completely manually broken and shortened with a metallographic saw to a size suitable for observation in an electron microscope. An example of a view of the analyzed sample is shown in Figure 5, where the level of completion of the sample fracture (the top skin during testing) is highlighted in red.

After evaluating the preliminary phase and identifying critical issues, new specimens were manufactured with improved input conditions, whose behavior under bending loading was experimentally investigated under the same conditions as the specimens in the first preliminary phase.

#### 3.3.2. Failure Load and Nominal Bending Stress

The failure load, as an ultimate force *f_u_* (n), was identified directly from the experimental load–deflection response as the maximum recorded load prior to a significant drop in force. This peak corresponds to the onset of global structural failure, which, in porous or architected materials, typically arises from progressive cell collapse, localized buckling, or fracture of load-bearing struts.

The bending stress in the specimens was evaluated using a nominal (engineering) formulation based on classical beam theory. The nominal bending stress (MPa) represents an engineering quantity derived from the global equilibrium of the beam and the assumption of a linear stress distribution in the cross-section [29,30]. Under three-point bending with a span length *L*, the maximum bending moment *M*_max_ occurs at mid-span, and the corresponding nominal bending stress can be calculated as [30]:

(5) $$\sigma_{\mathrm{nom}}=\frac{M_{max}}{W}=\frac{F_{u}L}{4W}\;\;\left(MPa\right),$$

where *W* denotes the section modulus of the specimen and *F_u_* is applied ultimate load.

This definition provides a consistent measure of the maximum apparent stress sustained by the structure under bending. The nominal bending strength *σ*_nom_ reflects the global load-carrying capacity of the architected structure, which is governed by its cellular topology, relative density/volume fraction, and deformation mechanisms; however, for porous AlSi10Mg, this does not represent the true material strength of the solid phase. It serves as a structural performance metric rather than a material property that is primarily used for comparing different porosity levels, architectures, or manufacturing conditions.

**Figure 6:**
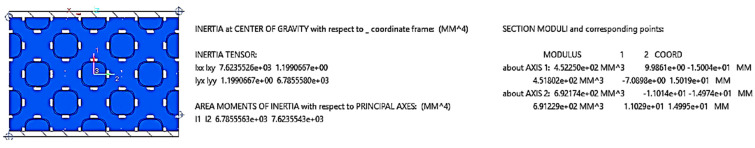
An example of the calculated result of the cross-sectional moment of inertia *I* and the section modulus *W* in a given sample of Schoen F-RD, *V_f_* = 10%.

Within this study, the section modulus of specimen *W* was taken from the characteristics of the 3D models of individual porous structures, where the section modulus was determined from the cross-sectional moment of inertia *I* according to *W* = *I*/*y*_max_, with *y*_max_ being the distance from the neutral axis to the most distant point of the cross-section. The corresponding *I* values were obtained from the complete virtual cross-section of each specimen, including both the TPMS cellular core and the upper/lower continuous skins, as shown in [22], since all of these parts contribute to the global bending resistance of the beam. An example of the calculated result of *I* and *W* in a given sample (specifically, in the case of Schoen F-RD with a volume fraction of 10%) is shown in Figure 6, in which the main axes 1 and 2 are also displayed.

**Figure 7:**
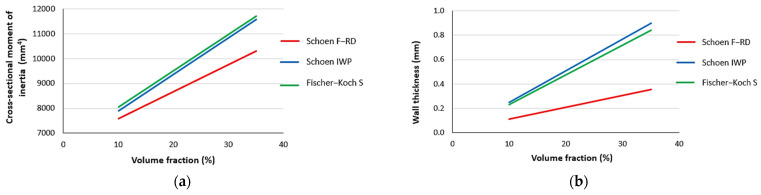
The dependences of (**a**) the wall thickness and (**b**) the moment of inertia on the volume fractions for individual types of investigated structures.

Although the virtual geometry accurately reflects the intended design and the use of the resulting section modulus provides a reliable standard for comparing specimens with different porosity levels or structural arrangements, small differences may occur between the virtual model and the actual fabricated specimens due to surface roughness, local porosity variations, and manufacturing process imperfections. To further specify the nominal stresses, wall thickness measurements were performed on preliminary fabricated structures, and the nominal *W* values were recalculated from these measurements. The dependences of the wall thickness and the moment of inertia on the volume fractions for individual types of investigated structures are plotted in Figure 7.

The computational procedure used to determine these integral geometric characteristics had been previously verified by the authors through analytical calculations on selected bodies and comparison with software-based numerical solutions [31], which supports the reliability of the adopted methodology.

#### 3.3.3. Effective Flexural Stiffness and Energy Absorption Evaluation

The effective flexural stiffness *k* of the cellular AlSi10Mg beams was evaluated from the initial linear portion of the load–deflection response obtained in three-point bending, within which holds d*F* = *k*d*w*. Within this region, deformation is governed by elastic bending of the intact cell walls, while the slope of the response reflects the undamaged structural stiffness. Based on the measured load–deflection data, the elastic region was determined as the interval satisfying the criterion of an approximately linear functional dependence, and this interval was subsequently evaluated by linear regression analysis. In this approach, stiffness was defined by the slope of the fitted linear function, i.e., the regression line representing the initial linear part of the response before the onset of visible non-linearity associated with local instability, damage initiation, or progressive structural failure.

To enable consistent comparison between specimens with different porosity levels or geometrical configurations, the energy absorption capability of the porous AlSi10Mg beams was quantified from the experimental load–deflection response as the area under the load–deflection curve using Equation (6) [32]:

(6) $$U(w)=\int_{0}^{w}F(\tilde{w})\;d\tilde{w},$$

The absorbed energy was evaluated directly from the measured force–deflection data by numerical integration of the experimental curve, up to a measured deflection limit *w*_max_ (mm), and the resulting quantity *U* (J) reflects the global energy dissipation capacity of the structure.

## 4. Identification of Critical Points of Failure Related to Production Settings and Testing

In the first phase, preliminary samples were fabricated from metal powder with a particle size range of 40–80 µm and a narrow Gaussian distribution around the diameter of 56 µm, as defined by the manufacturer. Based on the manufacturer’s recommendations for the samples, the height of the deposited layer was chosen to be 30 µm considering the uniformity and thin wall of the structures; likewise, the scanning strategy chosen was Meander with 90° interlayer rotation. Other 3D printing parameters were selected as follows: a scan speed of 2.5 m/s, a spot size of 70 µm, and hatch spacing set at 150 µm [33].

Several issues related to sample fabrication and testing were observed during the preliminary phase. Identifying these issues provided valuable insight into options for preventing premature sample failure.

### 4.1. Specification of Manufacturability and Testing Issues

**Figure 8:**
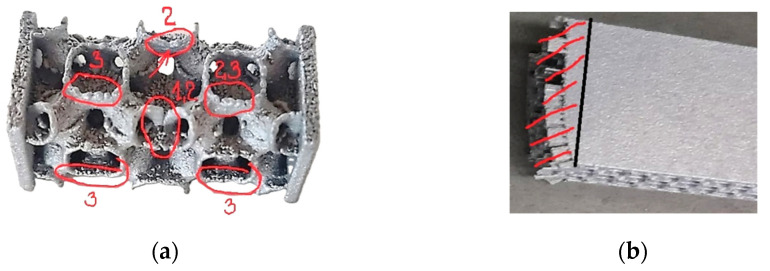
Failures in the 3D printing process during the preliminary phase; (**a**) failure issues encountered during the fabrication; (**b**) problem related to printing the last top layers.

One of the first problems that had to be solved in the production of porous samples was setting the minimum volume fraction in accordance with the capabilities of the 3D printer and the characteristics of the sample types, as well as the specified powder particle sizes. Multiple failure issues were encountered during the fabrication of samples at the lowest volume fraction, as illustrated in Figure 8a, which depicts a sample with *V_f_* = 8%. These were identified as:

- Overflows, collapses, and wall violations;
- Problems with self-supporting, i.e., capturing new layers on previous ones;
- Poor-quality frayed edges of cells, with a wavy character at the end;

as well as large amounts of residual powder, partially melted and stuck to the cell walls and bottom of samples.

After a few attempts with the fabrication of small samples, a volume fraction of 10% was set as an acceptable minimum for all three types of structures.

Another problem was related to printing the last top layers of the samples at their ends (Figure 8b), when the sample was positioned in the 3D printer workspace such that the skins of continuous material were parallel to the build platform. The proposed cutting off of the end parts could not be accepted because it would shorten the overall length of the sample, which would change the test conditions.

**Figure 9:**
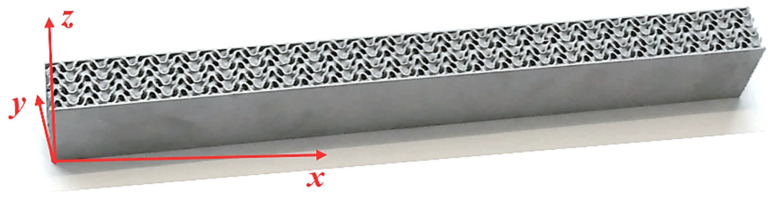
Position of the sample relative to the coordinate system of the workspace of the 3D printer.

Due to this, the samples were rotated 90 degrees so that the skins (layers of continuous material covering the cellular structure) were perpendicular to the platform (*xy* plane), as shown in Figure 9, while the *z* axis corresponds to the layering direction.

The position of the samples were also modified by rotating samples around the *z* axis (correlated with the layer deposition direction) by a small angle relative to the “*x*” axis during 3D printing due to the interaction of the recoater with the fine structure, so that there was gradual contact of the recoater with the sample (not impact) during the application of a consistent, uniform layer of powder material.

The samples were attached to the building platform by means of supporting structures to allow the samples to be removed from the platform with the desired sizes and without damaging the cellular structures. In this stage, the samples underwent stress-relief heat treatment at 285 °C in a convection air furnace while remaining on the build plate, held at this temperature for 2 h, followed by air cooling at rates equivalent to standard air cooling procedures, complying with ASTM F3318-18 [27,34]. This specification pertains to additively manufactured AlSi10Mg components (comparable to DIN EN 1706/EN AC 43000) [35] produced via powder bed fusion methods, such as laser melting. Afterward, they were cut from the build platform using an electrical discharge machine, and any remaining support structures were removed by sandblasting.

**Figure 10:**
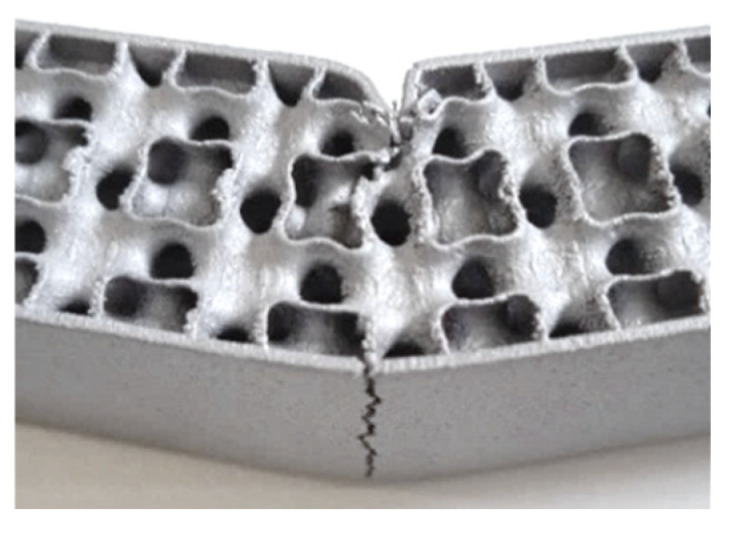
Damage in the top skin due to the pressure of the thumb of the testing machine.

During the testing of preliminary samples, the next problem with premature failure of the samples occurred (as shown in Figure 10) due to the small diameter (10 mm) of the pressure thumb of the testing machine, which, as it turned out, was inappropriately selected for the first tested sample. For all subsequent samples (preliminary as well as main—to evaluate flexural properties), a pressure thumb diameter of 20 mm was used for the second phase of investigation.

**Figure 11:**
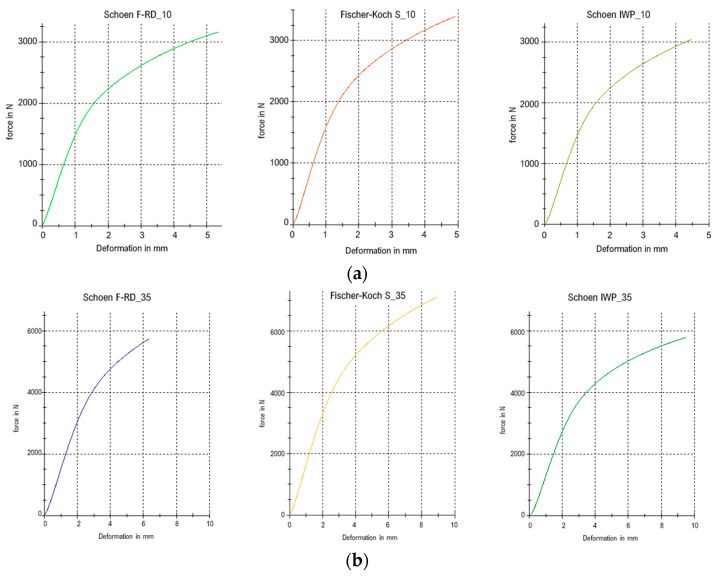
Graphs plotted during testing: (**a**) force–deflection dependences of preliminary samples with *V_f_* = 10%; (**b**) force–deflection dependences of preliminary samples with *V_f_* = 35%.

Initial samples underwent bending tests, and their force–deflection dependences are illustrated in Figure 11a (for *V_f_* = 10%) and Figure 11b (for *V_f_* = 35%). The bending nominal stress of individual specimens was calculated based on the moments of inertia of the nominal area for each structure topology. For specimens with a volume fraction of 10%, the values ranged from 230 MPa (Schoen IWP_10) to 258 MPa (Fischer–Koch S_10) and specimens with *V_f_* = 35% ranged from 317 MPa (Schoen IWP_35) to 342 MPa (Fischer–Koch S_35).

The results indicated the potential for improving mechanical properties with regard to the input parameters; therefore, further analyses related to the fracture surface were conducted.

### 4.2. Fracture Surfaces Evaluation

**Figure 12:**
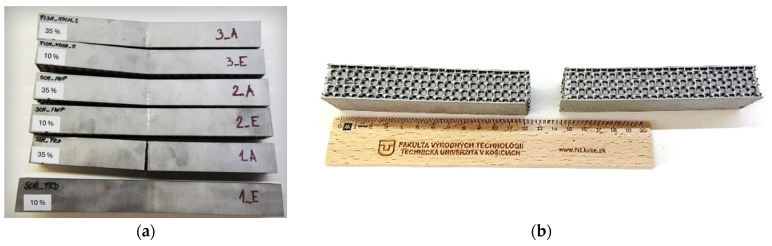
(**a**) A set of preliminary tested samples; (**b**) a representative Schoen F-RD_35 sample.

Six prefabricated samples with three types of structures (1—Schoen F-RD, 2—Schoen IWP, and 3—Fisher–Koch S; Figure 12a), each with a volume fraction of 10% (for “E” type samples) and 35% (for “A” type samples), were subjected to fracture surface analysis after preliminary experimental testing in bending. Representative findings regarding fracture surface analyses of prefabricated specimens are further presented in the analysis results of Shoen F-RD_35 specimen shown in Figure 12b.

**Figure 13:**
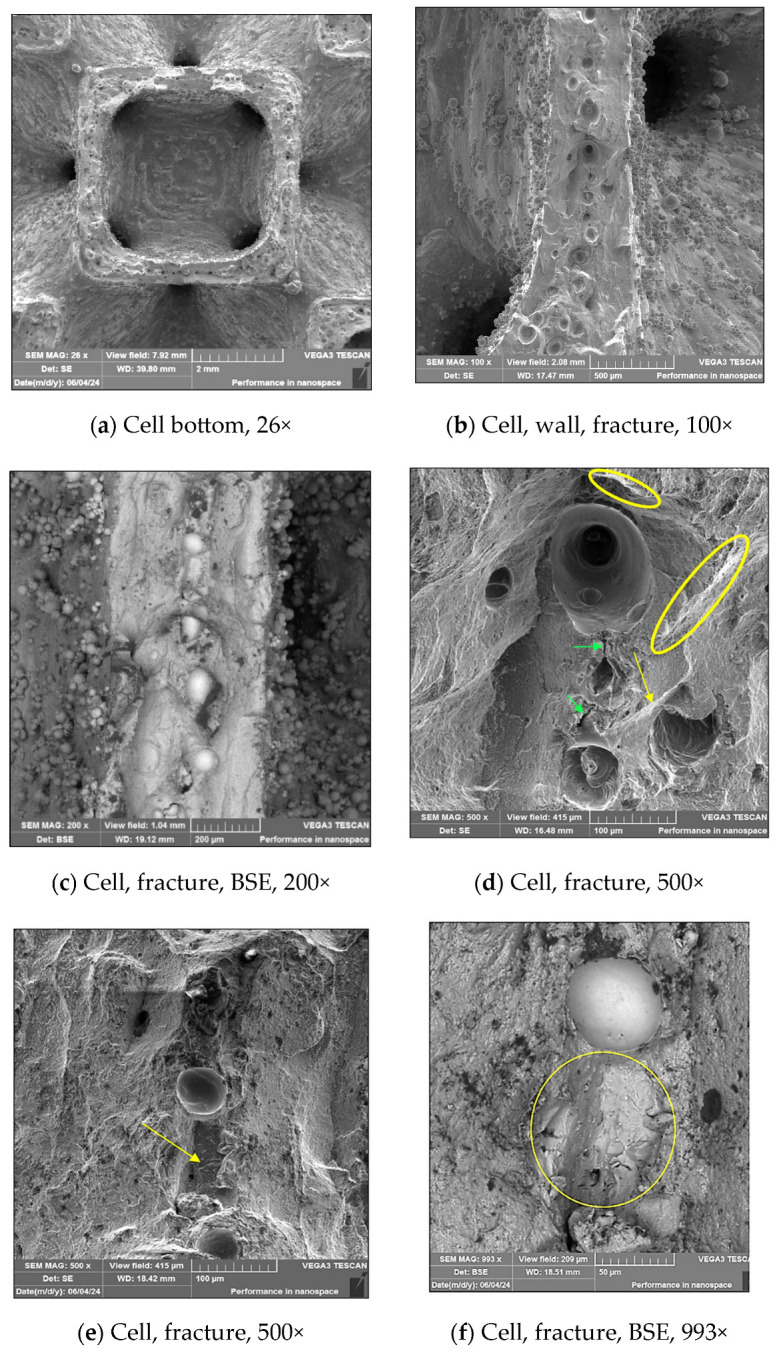
Fracture surface of sample 1A (Schoen F-RD structure with V*_f_* = 35%).

The cell wall of the Schoen F-RD structure with *V_f_* = 35%, its cell bottom, and the adjacent wall with particles on the surface are documented in Figure 13. As can be seen, the cell bottom (Figure 13a) is characterized by a certain roughness, which is related to the crystallization of the molten powder [36]. Partially melted (irregular shapes) and unmelted powder particles (regular spherical shapes) are visible on the surface of this area. The fracture surface of the sample on the cell walls contains a number of pores and particles that are arranged in lines (Figure 13b).

Upon closer examination using a BSE analyzer (Brucker SW Esprit 19), it is evident that the surface mainly contains a number of round particles and pores, which indicates that the powder particles have not been completely melted (Figure 13c,f). In the area between two unmelted powder particles, a region can be observed, which is probably formed by uneven crystallization of the melting, causing heterogeneity of the structure. In general, any phases that are incoherent or semi-coherent and interconnected with the matrix reduce the resistance to crack initiation and propagation.

For the detail of the fracture surface, Figure 13d,e documents that this was formed by ductile failure with a local pitting morphology and pronounced plastically deformed ridges (examples of ridges marked in yellow), which occur around the cavities. Due to the fact that the particles and pores were arranged in a line, essentially located in the center of the cell wall, this caused cracks to be locally formed when loading the sample material, which then propagated in the bridge (material between the individual cavities) perpendicular to the direction of loading of the sample (green arrows, Figure 13d).

The investigation identified a predominantly low-ductility failure mechanism, manifested in addition to globules of unmelted input material and the formation of fine size pits, tear ridges, flat facets, pores, and voids, similar to those reported in [33,37,38].

**Figure 14:**
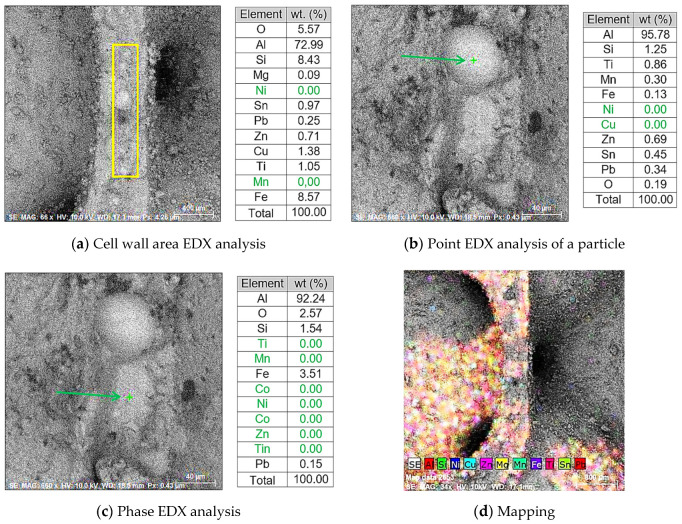
EDX fracture surface analysis and mapping—Schoen F-RD structure with V*_f_* = 35%.

EDX analysis was carried out on the fracture surface (presented in Figure 14a–c) and the distribution of elements, mapping this (Figure 14d) onto both the fracture surface and the adjacent wall.

The area EDX analysis, as shown in Figure 14a, documents the area selected for the chemical composition. The results contain elements that are related to the chemical composition of the powder material, as well as oxygen, which is typical for the fracture surface of Al-Si-based materials, because the newly created surface reacts with the atmosphere to form corrosion products of the Al_2_O_3_ type. Furthermore, a point EDX analysis of a selected particle of the fracture surface was performed (Figure 14b). The point analysis of the above-mentioned incoherent phase, as seen in Figure 14c, showed that this phase is relatively poor in chemical composition because it contains mainly Al, Si, and O, and, to a lesser extent, Mg, Mn, and Fe as well.

**Figure 15:**
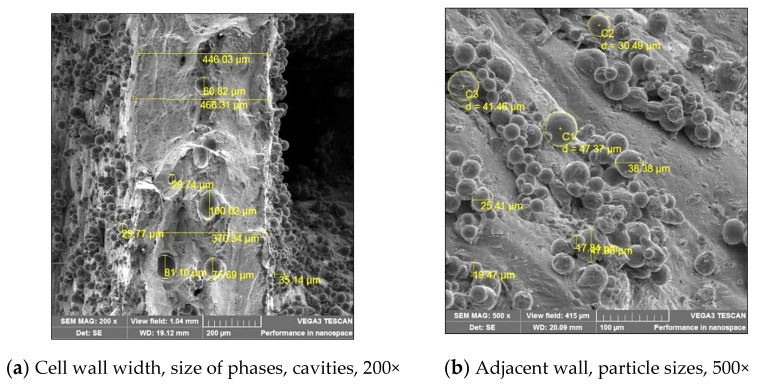
Fracture surface of the cell and adjacent walls, with measurement of their dimensions.

Another evaluation realized on the fracture surface was the determination of the width of the cell walls. These widths ranged from approximately 370 to 466 µm. The fracture surface also contained particles and pores, the dimensions of which ranged from approximately 30 to 100 µm, as seen in Figure 15a. A more detailed observation (Figure 15b) shows that these particles occur unevenly on the adjacent walls and form clusters. They are mostly spherical in nature, with dimensions ranging from 17 to 47 µm.

### 4.3. Discussion and Process Parameters Improvement

Preliminary examination of the samples pointed out some critical aspects related to 3D printing and testing of cellular structures as thin-walled objects with complex geometry. While some shortcomings could be relative easily eliminated (e.g., by rotating the sample by 90° during production or by replacing the pressure finger with a larger diameter), the defects in the cellular structures themselves needed to be analyzed in more depth, since the aluminum alloy AlSi10Mg is one of the most commonly used materials for 3D printing (SLM/LPBF), but its mechanical properties change significantly depending on the process parameters [39,40,41].

Given that, in this case, thin-walled structures were subjected to bending stress, the selection of appropriate process parameters was of fundamental importance, not only for the resulting mechanical properties but also for the qualitative properties, density, and internal stresses.

Based on the fracture surface analyses, it was possible to conclude that EDX analyses did not show any abnormalities, and the alloy composition corresponds to the one defined by the used material sheet. The fracture surfaces of all specimens were formed by ductile failure with a dimple-like morphology. The initiators of the failures were:

- Unmelted or partially melted metal powder particles, which were, in most cases, arranged in rows near the center of the cell;
- Cavities with residues of unmelted powder particles;
- Imperfectly interconnected material layers.

A large number of unmelted particles is a consequence of inappropriate conditions during the SLM process, which, in addition to the laser beam energy, includes the TEM (Transverse Electromagnetic Mode) of the laser, the laser beam speed, and its inclination. Subsequently, parameters such as layer height, hatching pitch, or the rotation angle between layers affect the energy delivered to the samples [42,43].

Under stable conditions of laser and 3D printing parameter settings during a single work cycle, the size of these particles had a major impact on whether the particles melted or not. It was likely that larger particles were not heated enough to melt (the total energy input required to heat them to the melting temperature was not sufficient). From an overall perspective, it could also be stated that the wide range of powder particle diameters resulted in some small particles burning and some not melting under the given setup conditions. So, the morphology and composition of the powder particles are among the critical factors affecting the quality of 3D-printed metal components. The consequences of these factors were the inhomogeneity and non-compactness of the material, increased porosity and lower density, insufficient fusion of melts resulting in potential microcracks between hatch lines, low ductility, the formation of cavities, and the weakening of the walls in cellular structures. Therefore, the input parameters were adjusted.

First, the energy supplied by the laser beam during production of the preliminary specimens was calculated. When expressing the laser energy for thin-walled components, it was appropriate to consider the so-called volumetric energy density (*VED*), which follows as [44]

(7) $$VED=\frac{P}{v\;h\;t}\;\;\;\;\;\;(J/{mm}^{3}),\;\;\;\;\;\;\;$$

where *P*—laser power (W); v—scanning speed (mm/s); h—hatch spacing (mm); and t—layer thickness (mm).

The initially selected process parameters (*P* = 500 W; v = 2500 mm/s; h = 0.15 mm; t = 0.03 mm), based on which the resulting *VED* value of 44.4 J/mm^3^ was determined, confirmed that these were not optimally chosen, since for the AlSi10Mg alloy and thin walls, the energy density interval proved to be 50–68 J/mm^3^ [45].

The observations during production parameter setup confirmed that, at this low energy density, a fine-grained celloid microstructure of Al + Si eutectic is formed, though not completely homogeneous, and after heat treatment (300 °C/2 h) → precipitation of β” (Mg_2_Si) is insufficient, because the time and temperature are low for a significant reinforcement reaction, as also noted by [46,47].

For the aim of improving the parameters and trying to prevent premature failure of the samples under bending stress, we therefore increased the *VED* so that the value was in the range of 55–65 J/m^3^, which, while maintaining the laser power of 500 W and the height of the deposited layer of 30 µm, was possible by reducing the scanning speed from 2.5 to 2 m/s or by reducing the hatching pitch from 150 to 120 µm. In both cases, the calculated volumetric energy density is 55.6 J/m^3^ caused higher Si diffusion during cooling, which resulted in a finer eutectic network and a more uniform Mg_2_Si distribution. The experiments revealed that for thin-walled components, it was more appropriate to reduce the scanning speed than to reduce the hatch spacing, because reducing the speed gave smoother energy and better homogeneity of the melt; moreover, when reducing the hatch spacing, there could be a risk of overheating the thin walls.

By combining the changes in the above parameters, or by reducing the height of the applied layer, the supplied energy value would already be high, and the metal powder would be burned through. However, the use of finer powder appeared to be a suitable adjustment of the input parameters and an alternative solution to reducing the layer height. Using a grain size range of 25–60 µm with a mean value of 45 µm ensured more suitable absorption of the delivered energy, better surface quality, and a finer microstructure. Changing the scanning strategy from Meander with 90° rotation to Chessboard by limiting the scan length to 5 × 5 mm and rotation to 67° helped to reduce the stress and strains in the thin walls of the structure.

It turned out that the heat treatment used for the samples tested in the first phase was only suitable for removing internal stress. To improve the mechanical properties and balance the combination of strength + ductility, the heat treatment for removing internal stresses (performed at 300 °C/2 h in a conventional air furnace with subsequent cooling in air) was supplemented with natural aging for 24 h, solution annealing with continuous heating to 530 °C, holding at this temperature for 1 h, immediate quenching in water, aging at 170 °C for 6 h, and then followed by cooling in air [27].

A comparison of the process parameters used for the first (preliminary) and second (main testing) phases of sample production are shown in Table 2.

**Figure 16:**
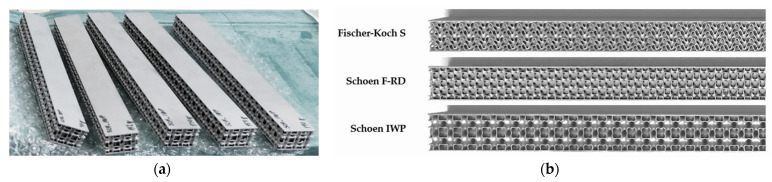
Examples of sets of produced samples: (**a**) a set of samples of the same type of structure (Schoen IWP) with five different volume fractions; (**b**) a set of samples of the same volume fraction of 10% with different types of structures.

To ensure the repeatability of the test, three specimens of the same type (given by the same cell design and volume fraction) were manufactured, so 45 samples were produced for testing in the main phase in total. A set of samples with the same type of structure (Fischer–Koch S) but with five different volume fractions is shown in Figure 16a, and a set of samples with the same volume fraction of 10% but with different types of cellular structures is shown in Figure 16b.

## 5. Results and Discussions

### 5.1. Research Results

**Figure 17:**
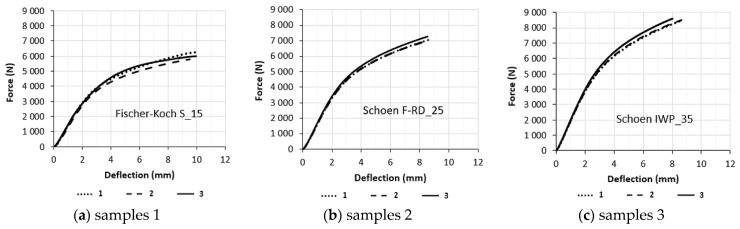
Example of the course of the recorded dependencies for three topologically identical samples.

The manufactured samples showed good agreement in terms of repeatability when testing three identical samples 1, 2, and 3 with the same topology (Figure 17), which was also confirmed by statistical data processing, with no sample being excluded from further evaluation due to outliers.

The Grubbs test was used as part of the statistical processing to find outliers. For each type of sample, mean values of individual specific characteristics were calculated, with the standard deviation from this value for no characteristic exceeding 5%.

**Figure 18:**
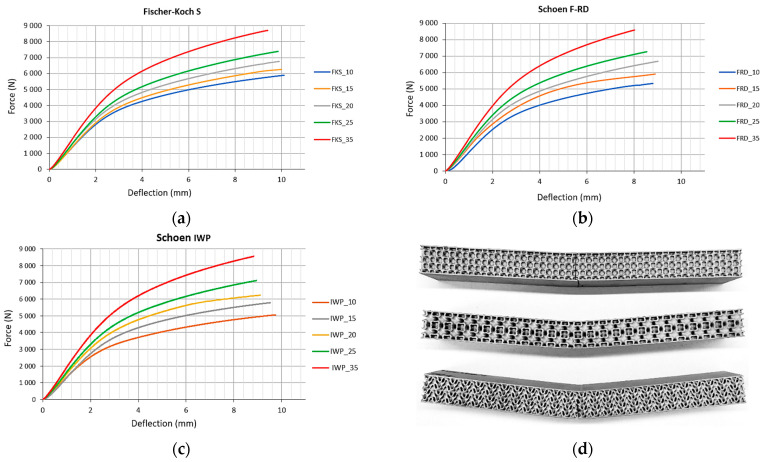
Representative load–deflection curves for five different volume fractions: (**a**) Fischer–Koch S; (**b**) Schoen F-RD; (**c**) Schoen IWP; (**d**) damaged samples of different structures with *V_f_* = 25%.

To determine the significance of the hypothesis, the critical *p*-value was also calculated at a significance level of α = 0.05, and linear models of sample behavior were specified by regression analysis, with the coefficient of determination R^2^ being higher than 0.985 in all cases, indicating a strong functional dependence. Representative force–deflection plots of the same structure with different volume fractions are shown in Figure 18a–c to allow for a first-sight comparison of their behavior during bending, while Figure 18d shows the damaged samples of different structures with the same volume fraction of 25%.

**Figure 19:**
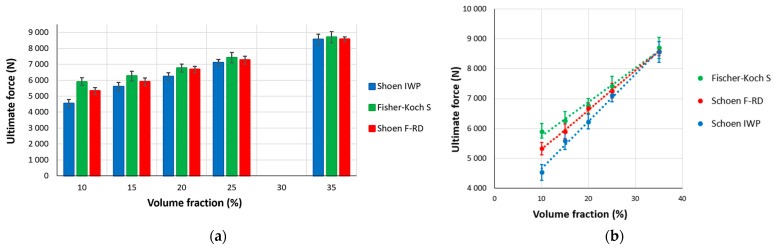
Measured maximum forces for individual structures: (**a**) overview comparing maximum forces at the same volume fraction; (**b**) trends of increasing values with increasing volume fraction.

A comparison of the measured ultimate forces required to damage the beams with cellular structures for individual topologies is shown in Figure 19a, while a closer look at the trends of increasing in average values of maximal forces is shown in Figure 19b.

For the research area with a sample volume fraction range of *V_f_* = 10–35% under the above conditions, the functional dependences of ultimate force (N) on volume fraction *V_f_* for individual structures were defined as follows; however, the continuation of this trend at higher *V_f_* is questionable and requires further investigation.

Fischer–Koch S (R^2^ = 0.9892):

*y* = 114.29*x* + 4603.3 (8)

Schoen F-RD (R^2^ = 0.9989):

*y* = 130. 82*x* + 4001.5 (9)

Schoen IWP (R^2^ = 0.9954):

*y* = 158.42*x* + 3073.7 (10)

**Figure 20:**
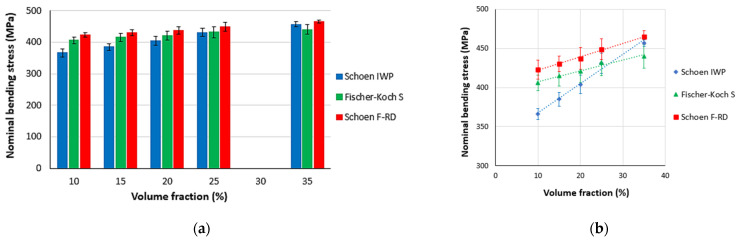
Evaluated nominal bending stress of individual structures: (**a**) overview comparing strength at the same volume fraction; (**b**) trends in increasing strength values with increasing volume fraction.

Regarding the nominal bending stresses (Figure 20a), due to the different geometry of the structures, the trend of increase with volume fraction (Figure 20b) is different compared to forces.

The dependences of nominal bending stress (MPa) on volume fraction *V_f_* can be expressed using the following equations:

Fischer–Koch S (R^2^ = 0.9865):

*y* = 1.3595*x* + 393.85 (11)

Schoen F-RD (R^2^ = 0.9931):

*y* = 1.7189*x* + 404.7 (12)

Schoen IWP (R^2^ = 0.9856):

*y* = 3.7351*x* + 330.16 (13)

The highest average nominal values of the bending stress at all evaluated volume fractions were shown by Schoen F-RD, with an achieved value of 465 MPa at a volume fraction of 35%, slightly higher than the Fischer–Koch S structure of 440 MPa. Nevertheless, these differences should be interpreted as relatively small in structural terms and not as evidence that one topology is categorically superior in all evaluated characteristics.

Comparison of nominal bending stress values of preliminary samples and samples produced in the second phase indicate a significant influence of technological factors on mechanical properties, as stresses in comparable samples with the same topology were at least 100 MPa higher in the second phase, with larger differences observed in samples with the lowest volume fraction *V_f_* = 10%; for example, for the Schoen IWP structure, there was an increase from 230 to 366 MPa, while for Fischer–Koch S, from 258 to 406 MPa.

**Figure 21:**
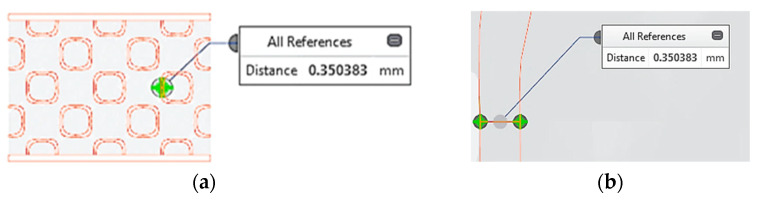
Analysis of the wall thickness of a 3D model: (**a**) view of the cross-sectional area of the model at the measurement location; (**b**) measurement detail.

However, since the measured wall thicknesses of the structures in the fracture surfaces (for example, for the Schoen F-RD structure, these were measured from 370 to 466 µm) were higher compared to the nominal values of the created 3D virtual models at a given location of the cell (that is, e.g., for Schoen F-RD 350 µm, Figure 21), it should be taken into account that the corrected nominal bending stress values are reduced by approximately 5–10%; therefore, with the maximum achieved (average) value of 465 MPa for the Schoen F-RD_35 structure, this represents a reduction in the nominal stress value to the range of 440–420 MPa.

**Figure 22:**
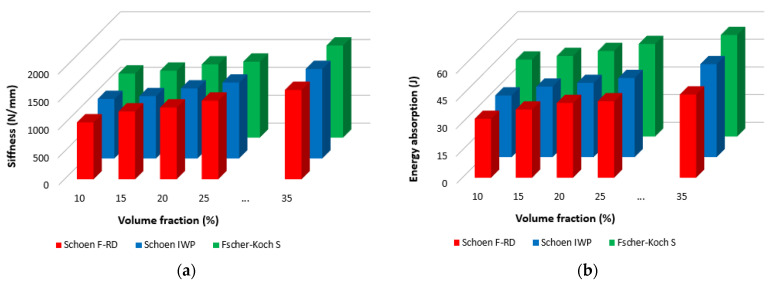
Evaluated flexural properties: (**a**) stiffness in elastic region; (**b**) total amount of absorbed energy until failure.

Similarly, as the maximum force achieved increases with volume fraction, the flexural stiffness of the structures in the elastic region (Figure 22a) and the amount of total energy absorbed to failure (Figure 22b) increase, with the Fischer–Koch S structure showing the highest mean values among the evaluated structures, which were in the range of 1150–1655 N/mm and 42–55 J. 

Specific values of stiffness and energy absorption are provided in Table 3 and Table 4, respectively. This indicates that, within the investigated benchmark configuration, the Fischer–Koch S topology exhibited the most favorable balance of stiffness and absorbed energy, whereas the Schoen F-RD topology tended to reach slightly higher nominal bending stresses.

The values will vary with component dimensions and volume fractions, but a comparative study of experimental testing has shown that, although the differences in mechanical properties between the structures are not extreme, there are still relative differences in the behavior of the structures at a given volume fraction, with the Fischer–Koch S structure appearing to demonstrate the most balanced properties within studied types of samples.

To compare the tested group of samples regarding ultimate forces, nominal stresses, stiffnesses and energy absorptions from a statistical point of view, one-way ANOVA and Tukey HSD tests were performed. The results of all tests have been in the 95% region of acceptance: [0:3.8853] with a *p*-value greater than the standard 0.05 significance level so the difference between the sample averages of all groups is not big enough to be statistically significant. Corresponding F and *p*-values are listed in Table 5. However, the observed effect size f (η^2^) indicates that the magnitudes of the differences between the averages are different—for ultimate forces, this is medium; nominal stresses is large; stiffness is small; and energy absorption is large.

**Figure 23:**
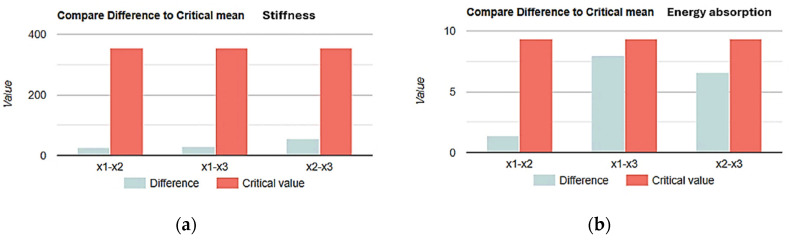
An example of comparison differences to critical means for evaluated data of (**a**) stiffness and (**b**) energy absorption.

Tukey HSD tests, at all statistically evaluated values, confirmed that there were no significant differences between the means of any pair. Figure 23 shows examples of comparison differences to critical means for evaluated data of stiffness and energy absorption.

### 5.2. Discussion

Investigation of preliminary produced samples and the observed fracture morphology indicated that the failure cannot be interpreted as purely brittle crack propagation. Rather, the results are more consistent with a predominantly ductile failure mode with reduced local plasticity, in which crack initiation was facilitated by process-related defects, such as unmelted or partially melted powder particles, cavities containing powder residues, and imperfect interconnection of material layers. The locally observed pits, plastically deformed ridges, pores, and flat facets suggest a combined fracture character at the microscale; however, these features do not support classification as a fully brittle mechanism throughout the fractured section. In addition, the measured wall thickness range of approximately 370–466 µm, together with pores/cavities in the range of approximately 30–100 µm and particle clusters in the range of approximately 17–47 µm observed on representative fracture regions, indicates that defect dimensions were not negligible relative to the local wall thickness and could therefore substantially affect crack initiation in the tensile zone of bent specimens.

The improvement in bending-related properties is most likely the result of the combined action of several modified processes and postprocessing parameters. Because these parameters were changed simultaneously, the present experimental design does not allow a quantitative decoupling of their individual effects. However, a qualitative comparison suggests that the largest contribution is likely associated with parameters that directly control melt pool formation and overlap, namely scan speed, hatch spacing, layer thickness, and powder-size distribution. These variables strongly influence local energy input, melt pool stability, intertrack and interlayer bonding, and consequently the formation of lack-of-fusion defects and residual porosity, which are among the most direct factors governing strength and stiffness in LPBF-built AlSi10Mg. Scan strategy and 67° layer rotation likely play a secondary, though still important, role by modifying the thermal history of successive layers, reducing the repetition of scan tracks, and thereby contributing to a more uniform distribution of thermal gradients, residual stresses, and anisotropic features. Their primary effect is therefore expected in the reduction in localized defect accumulation and in the stabilization of the overall mechanical response rather than in the direct densification mechanism itself.

Finally, the postprocessing heat treatment is expected to act mainly through microstructural evolution rather than through the initial creation of density. While stress relief primarily reduces internal stresses, solution treatment followed by aging can alter the morphology and distribution of Si-rich phases and influence hardness, ductility, and crack-initiation sensitivity in AlSi10Mg [48]. For this reason, the improved bending response observed in the present work is best interpreted as a synergistic outcome of energy input-related parameters, scan-path design, and heat-treatment-induced microstructural modification, rather than as the effect of one isolated dominant variable.

A qualitative interpretation of the measured response of the investigated structures suggests that the Fischer–Koch S topology provides a more continuous load-transfer path and a more uniform participation of the cell walls in bending deformation, which is consistent with its higher stiffness and greater energy absorption. In contrast, the Schoen F-RD topology appears to sustain slightly higher nominal bending stresses, possibly due to a more favorable local stress-bearing configuration in the critical bending region, although this may also be associated with stronger stress concentration and earlier deformation localization. More generally, the observed response of the tested sandwich-type specimens is interpreted as a combination of global beam bending, local bending/stretching of thin TPMS cell walls, skin–core interaction, and tensile-side fracture initiation in defect-weakened regions; localized wall instability or local buckling may also contribute before final fracture.

Further studies, which the authors would like to carry out in the near future, should not only confirm the findings but also examine the behavior of the structures in more detail in terms of other properties, formalizing the influence of individual factors from a mathematical and numerical perspective in order to predict the behavior of the structures in real applications; moreover, the implementation of scientific and physical principles with the authors’ already published patent claims [49,50] will also play a significant role in the investigation.

## 6. Conclusions

The article aimed to study the bending behavior of three selected complex triply periodic porous structures (Schoen IWP, Fischer–Koch S, and Schoen F-RD) made of AlSi10Mg alloy via the SLM method. Within the presented comparative study, the flexural behavior of the sandwich-type specimens with five different volume fractions of 10, 15, 20, 25, and 35% (±1%) were investigated. As part of the preparatory phase, samples with the smallest and highest selected volume fractions were produced, while analyzing the critical root aspects of not only the manufacturability of thin-walled structures with complex shapes but also premature failure during testing. The combined use of preliminary manufacturing optimization, bending experiments, and fracture surface analysis made it possible to relate the flexural response of the investigated TPMS topologies to process-induced defects and their effect on premature failure in thin-walled AlSi10Mg cellular structures.

- The analysis performed in the preparatory phase revealed that the fracture surfaces of all examined samples were formed by ductile failure with dimple morphology. The survey identified a predominantly low-ductility failure mechanism, manifesting in addition to globules of unmelted input material and the formation of fine pits, cracks, flat facets, pores, and voids.
- Analysis of fracture surfaces showed that the wide range of powder particle diameters resulted in some small particles being burned and some not being melted, indicating improperly set input manufacturing parameters and conditions. The regions between the unmelted powder particles caused structural heterogeneity, while the incoherent or semi-coherent bonding of the unmelted particles to the matrix reduced resistance to crack initiation and propagation. The particles/pores arranged in a line, essentially located in the center of the cell wall, caused local cracks to form when the material sample was loaded, which propagated in the bridge (material between the individual cavities) perpendicular to the direction of loading of the sample.
- By changing the energy value of the laser beam supplied into the alloy powder material during production—namely by reducing the scanning speed from 2.5 to 2 m/s and by changing other input factors, such as using a finer powder or changing the scanning strategy with a subsequent change in the heat treatment process—the samples were able to carry much higher loads, and the nominal stresses in comparable samples with the same topology in the second phase were at least 100 MPa higher than in the samples produced in the first phase.
- The highest average nominal values of bending stress at all evaluated volume fractions were shown by Schoen F-RD, with a value of 465 MPa at a volume fraction of 35%, which is slightly more than the Fischer–Koch S structure with 440 MPa. At the same time, the Fischer–Koch S topology showed the highest values of bending stiffness in the elastic region and total energy absorbed until failure among the evaluated structures, which ranged between 1150–1655 N/mm and 42–55 J, respectively, indicating a more balanced flexural response within the investigated benchmark configuration.

The presented study pointed out the potential of selected TPMS structures for lightweight core concepts, but at the same time, it highlighted the challenges associated with the technology and production of thin-walled, complex-shaped elements. The results were obtained for one material system (SLM-manufactured AlSi10Mg), one specimen geometry, and one loading mode (quasi-static three-point bending), and they provide a benchmark basis for comparing flexural response, manufacturability-related defects, and topology-dependent failure behavior. Broader validation of these structures for engineering components subjected to combined bending–torsion loading, fatigue, contact, or dynamic effects will require further studies using more representative loading cases, broader quantitative defect population analysis, and FE-based mechanistic modeling.

## Figures and Tables

**Table 1:** Chemical composition of AlSi10Mg alloy [27].

Element	Al	Mg	Si	Ni	Sn	Pb	Cu	Zn	Ti	Mn	Fe	S
wt. (%)	Bal.	0.2÷0.45	9÷11	<0.05	<0.05	<0.05	<0.05	<0.1	<0.15	<0.45	<0.55	<0.05

**Table 2:** Comparison of process parameters of sample production in individual phases.

Parameter	Preliminary (First) Phase	Main (Second) Phase
Laser power	500 W	500 W
Spot size	70 µm	70 µm
Layer thickness	30 µm	30 µm
Metal powder	40–80 µm	25–60 µm
Scan speed	2.5 m/s	2.0 m/s
Hatch spacing	150 µm	150 µm
Scanning strategy	Meander, 90° rotation	Chessboard (island 5 × 5 mm), 67° rotation
Heat treatment	300 °C/2 h	300 °C/2 h; 530 °C/1 h + 170 °C/6 h

**Table 3:** Stiffness (N/mm).

*V_f_* (%)	Structure Type		
	Schoen F-RD	Schoen IWP	Fischer–Koch S
10	1022 ± 33	1069 ± 37	1150 ± 39
15	1218 ± 48	1119 ± 45	1202 ± 52
20	1289 ± 56	1258 ± 47	1315 ± 61
25	1412 ± 63	1363 ± 56	1364 ± 61
35	1604 ± 69	1606 ± 72	1655 ± 70

**Table 4:** Energy absorption (J).

*V_f_* (%)	Structure Type		
	Schoen F-RD	Schoen IWP	Fischer–Koch S
10	32 ± 1.3	33 ± 1.3	42 ± 1.7
15	37 ± 1.5	38 ± 1.6	44 ± 1.7
20	41 ± 1.7	40 ± 1.7	46 ± 1.9
25	42 ± 1.7	43 ± 2.0	50 ± 2.2
35	45 ± 2.0	50 ± 2.5	55 ± 2.5

**Table 5:** One-way ANOVA test results.

	F	*p*-Value	f	η^2^	Magnitude
Ultimate force	0.267293	0.769888	0.21	0.043	Medium
Nominal stress	2.211641	0.15217	0.61	0.27	Large
Stiffness	0.0834603	0.920457	0.12	0.014	Small
Energy absorption	2.98257	0.0888186	0.71	0.33	Large

## Data Availability

Data are contained within the article.

## Abbreviations

The following abbreviations are used in this manuscript:

TPMS	Triply Periodic Minimal Surface
SLM	Selective Laser Melting
